# A case series of prenatal hepatic hilar cyst in the presence of a gallbladder - navigating the dilemma between biliary atresia and choledochal cyst

**DOI:** 10.1186/s12887-024-05043-z

**Published:** 2024-09-13

**Authors:** Ana M. Calinescu, Anne-Laure Rougemont, Valérie A. McLin, Nathalie M. Rock, Céline Habre, Barbara E. Wildhaber

**Affiliations:** 1 Swiss Pediatric Liver Center, Department of Pediatrics, Gynecology and Obstetrics, University of Geneva, Geneva, Switzerland; 2 Division of Child and Adolescent Surgery, Department of Pediatrics, Gynecology, and Obstetrics, Geneva University Hospitals, University of Geneva, 6, Rue Willy Donzé, Geneva, 1205 Switzerland; 3 Division of Clinical Pathology, Diagnostic Department, Geneva University Hospitals, University of Geneva, Geneva, Switzerland; 4 Gastroenterology, Hepatology and Nutrition Unit, Division of Pediatric Specialties, Department of Pediatrics, Gynecology, and Obstetrics, Geneva University Hospitals, University of Geneva, Geneva, Switzerland; 5 Division of Pediatric Radiology, Diagnostic Department, Geneva University Hospitals, University of Geneva, Geneva, Switzerland

**Keywords:** Cystic biliary atresia, Choledochal cyst, Prenatal hilar hepatic cyst, Hepaticojejunostomy, Case report

## Abstract

**Background:**

Prenatally diagnosed hepatic hilar cysts are a challenging finding for the clinician. They can either be a sign of cystic biliary atresia (BA) or a choledochal cyst (CC), two diagnoses with different postnatal management and prognosis. Based on a case report of four patients, we aim to propose a management algorithm for prenatally diagnosed “hepatic hilar cysts”.

**Case presentation:**

A hepatic hilar cyst, ranging from 5 to 25 mm, was detected prenatally in all four girls confirmed postnatally along with the presence of a gallbladder. Stool color was normal until two weeks of life at which time the stool color became lighter, and the patients developed cholestasis. All were operated before seven weeks of life: Case 1 had a CC with patent but irregular intrahepatic bile ducts at intraoperative cholangiogram, and no communication with the duodenum. A Roux-en-Y bilioenteric anastomosis was performed. The cyst showed complete epithelial lining loss, and liver pathology showed BA features. Case 2 had the final diagnosis of cystic BA with patent but abnormal intrahepatic bile ducts. She underwent two operations: the first operation at four weeks as described for case 1, since intraoperative findings were similar, as was histology. As cholestasis increased postoperatively, she underwent a Kasai hepato-porto-enterostomy six weeks later, where distinct BA findings were found with complete scarring of the hilar plate. Case 3 had a cystic BA with the cyst located within the common bile duct and atretic bile ducts proximal to the porta hepatis. It exhibited no communication with the liver or duodenum. A Kasai operation was performed, with histology showing complete epithelial loss within the cyst wall and scarring of the hilar plate. Case 4 had a cystic BA presenting a completely obliterated hepatic duct with the cyst lying within the common bile duct. A Kasai procedure was performed. Histology showed a common bile duct with a residual lumen of 0.1 mm.

**Conclusions:**

The spectrum of disease from CC to BA in the setting of a prenatally discovered hepatic hilar cyst is emphasized. Even if cholangiogram differentiates most patients with BA from those with CC, caution is advised for transitional types.

## Background

Today it is generally acknowledged that prenatally diagnosed hepatic hilar cysts in the presence of a gallbladder have two distinct differential diagnoses, choledochal cyst (CC) versus biliary atresia (BA) [1] with a differential diagnosis of solitary liver cysts and gallbladder duplication. A hepatic hilar cyst is present in up to 16% of BA patients [2–5]. Two patterns of cystic BA can occur: most frequently they correspond to BA with patent but abnormal intrahepatic bile ducts and an extrahepatic cyst located within the hepatic duct or, more rarely, to BA with atretic bile ducts proximal to the porta hepatis and an extrahepatic cyst in the fibrous choledochal remnant [6–9]. CC also have distinct subtypes, whereby the typical form with the presence of a cyst in the common bile duct (Todani classification type I) accounts for up to 90% of CC [10].

In the setting of a prenatal hepatic hilar cyst, postnatal ultrasound (US) with Doppler is the first imaging examination to differentiate between a BA and a CC [11]. A hepatic hilar cyst with dilated intrahepatic bile ducts is diagnostic of CC and rules out BA. Nevertheless, in unclear presentations, i.e. a hilar cyst with abnormal gallbladder and/or triangular cord sign, an intraoperative cholangiogram (IOC) is warranted [12].

Patients with CC classically undergo cyst excision and hepatico-jejunostomy, BA patients undergo Kasai hepato-porto-enterostomy (HPE). While Kasai HPE should be performed as soon as possible, hepatico-jejunostomy for perinatally diagnosed CC can be differed in the first months of life, with the need for swift intervention in case of neonatal cholestasis [13]. Identifying the correct surgical approach and timing is essential for optimal patient outcome. While CC patients usually are definitely managed by hepatico-jejunostomy, cystic BA patients, even if endowed with higher native liver survival rates, still might need, at some time point, a liver transplantation [14].

The present case report illustrates the step-by-step process of clinical decision-making for patients prenatally diagnosed with hepatic hilar cysts in the presence of a visible gallbladder in the unique setting of four patients with transitional types of disease.

## Case presentation

Preoperative, operative, pathology and postoperative characteristics of four challenging female patients with a prenatally diagnosed hepatic hilar cyst diagnosed between 2020 and 2022 with a follow-up of 15.5–38 months were reviewed. The present study follows the guidelines of the revised UN Declaration of Helsinki and complies with the local regulations in the Geneva University Hospitals, Swiss Ethics (Ethics Committee no. AO_2022-00073). Informed consent was obtained from each patient’s legal guardian.

### Pre- and immediate postnatal evaluation

**Fig. 1:**
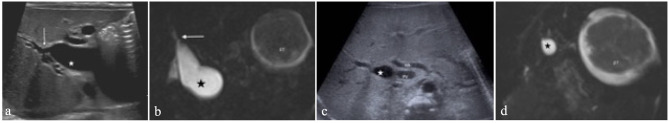
Ultrasound and magnetic resonance cholangiopancreatography coronal maximum intensity projection reformat. In Case 1, a large cyst of the liver hilum (stars) communicating with an intrahepatic bile duct (white arrow) at both ultrasound (**a**) and MRCP (**b**). In case 2, a smaller hilar cyst (stars) without any visible communication with intra- or extrahepatic bile ducts at both ultrasound (**c**) and MRCP (**d**). HA, hepatic artery; PV, portal vein; ST, stomach; MRCP, Magnetic resonance cholangiopancreatography

All four girls presented a hepatic hilar cyst with a prenatal size from 5 to 25 mm, measured at different time-points with US and magnetic resonance cholangiopancreatography (MRCP), not evolving over time. Liver stiffness was evaluated using acoustic radiation force impulse imaging (normal range 0.73–1.45 m/sec) [15]. Post-natal findings are summarized in Table 1; Fig. 1a, b, c, d. Overall, stool color was normal until two weeks of life when they progressively started to discolor, and the patients gradually became cholestatic, prompting surgery, all before seven weeks of life.

**Table 1:** Summary of the post-natal findings in the four patients

	Case 1	Case 2	Case 3	Case 4
Final diagnosis	Todani I Choledochal cyst	Cystic BA with patent but abnormal intrahepatic bile ducts	Cystic BA with atretic bile ducts proximal to the porta hepatis	Cystic BA with atretic bile ducts proximal to the porta hepatis
Sex	F	F	F	F
Birth (weeks)	38 3/7	41 3/7	40 4/7	37 3/7
Cyst size (mm) at 1st post-natal US	20	10	11	5
Icterus (days)	From birth	21	From birth	None
Preoperative maximum conjugated bilirubin (µmol/l)	98.9	81.3	101	63
Preoperative maximum GGT (U/l)	86	91	99	195
US gallbladder	Normal	Irregular wall	Small size Irregular wall	Small size Irregular wall
Hyperechoic triangular cord sign	None	None	None	Present
US bile duct dilatation	None	None	None	None
Liver stiffness m/sec	1.64	2.01	n.a.	1.26
MRCP	Hilar cyst communicating with right bile duct yet dilated	Non-communicating hilar cyst No dilated intrahepatic bile ducts	n.a.	n.a.
Intraoperative cholangiogram	Patent intrahepatic bile ducts No duodenal drainage	Patent intrahepatic bile ducts No duodenal drainage	No intrahepatic bile ducts No duodenal drainage	No intrahepatic bile ducts No duodenal drainage
Bile colour	Transparent	Transparent	Transparent	Transparent
1st Surgery	Cyst excision, hepatico-jejunostomy	Cyst excision, hepatico- jejunostomy	Kasai procedure	Kasai procedure
Age at 1st surgery (days)	42	28	55	42
2nd Surgery	n.a.	Kasai procedure	n.a.	n.a.
Age at 2nd surgery (days)	n.a.	69	n.a.	n.a.
Follow up (months)	38	28	15.5	32

### Operative findings

**Fig. 2:**
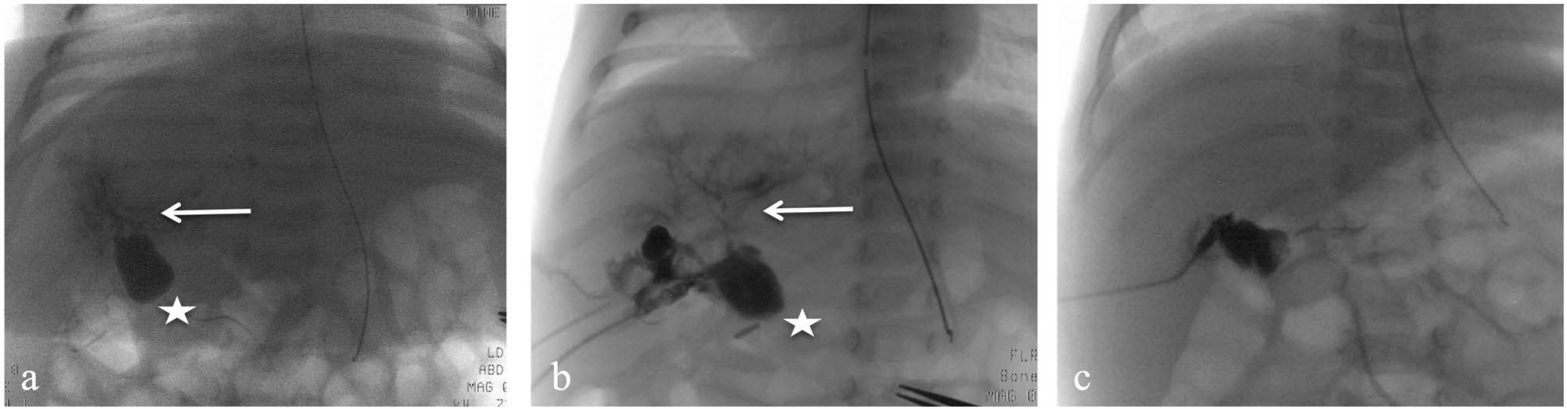
Intraoperative cholangiogram. Case 1 (**a**) and 2 (**b**) showed patent but irregular intrahepatic bile ducts (arrow) and no communication with the duodenum (star). Case 4 (**c**) showed no communication with either intrahepatic bile ducts or duodenum

In all four cases an intraoperative cholangiogram (IOC) via the gallbladder was performed (Fig. 2a, b, c): retrograde opacification of patent but thin and irregular intrahepatic bile ducts, with no opacification of the duodenum, was present in Cases 1 and 2 (Fig. 2). The fluid collected from the cyst was nearly transparent and contained no pigment. Amylase and lipase values in the cyst were normal. Inflammatory adhesions were mild around the cyst in Case 1 and moderate in Case 2. The liver surface was irregular in Case 1 and the liver soft, while it was smooth and soft in Case 2. Given the presence of a clear communication of the cyst at the level of the hepatic duct with the intrahepatic bile ducts, a Roux-en-Y bilio-jejunal anastomosis was performed. Cases 3 and 4 showed absence of retrograde filling of contrast in the intrahepatic bile ducts reflecting their complete obliteration (Fig. 2), with the hilar cyst lying within the common bile duct, corresponding to a cystic BA with atretic bile ducts proximal to the porta hepatis, consequently a Kasai HPE was performed. The liver surface was smooth and the liver firm for Cases 3 and 4.

**Fig. 3:**
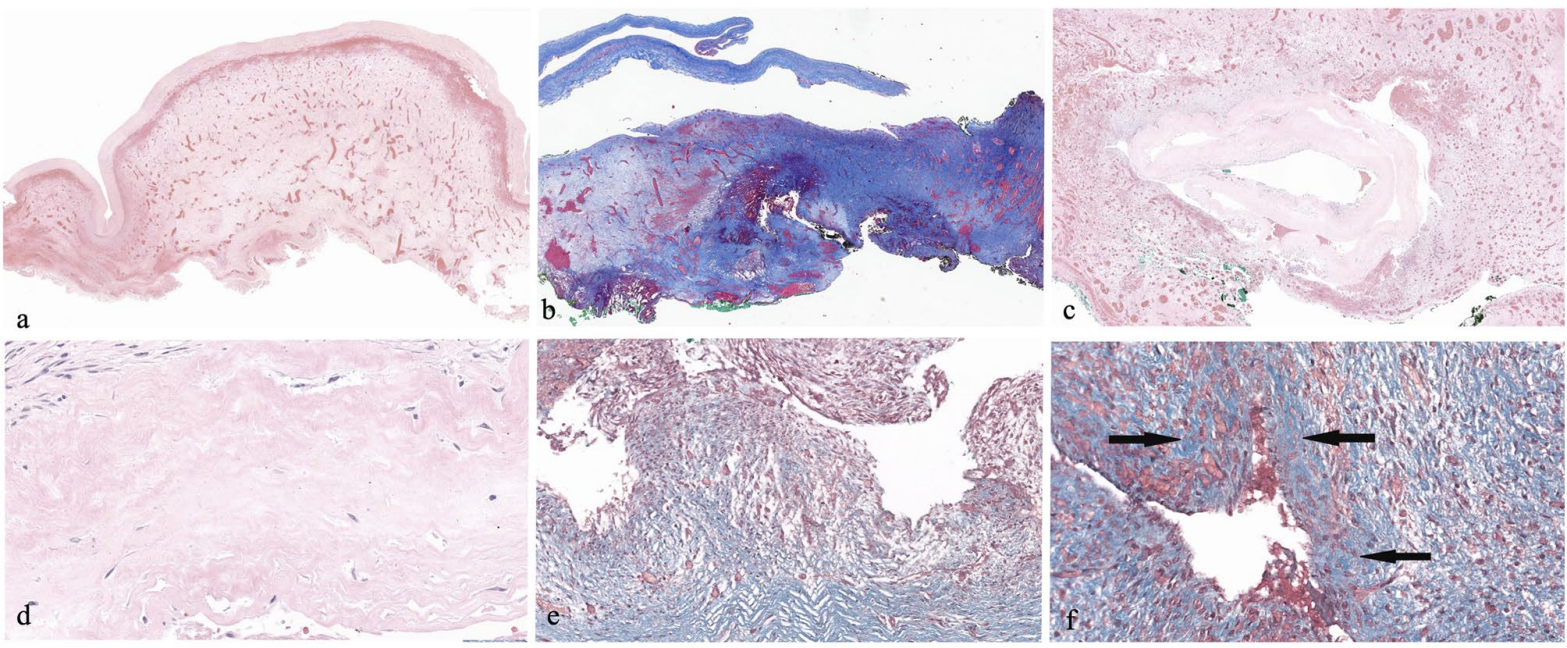
The cysts from Cases 1 to 3 (**a** to **d**) showed similar histological features, whereas findings were different in Case 4 (**e** and **f**). **a**. In Case 1, the 3.5 cm long cyst showed no biliary epithelium; instead, the luminal portion of the cyst consisted of a dense hyaline layer overlying a congestive outer cyst wall (Hematoxylin& Eosin, H&E, original magnification x60). **b**. In Case 2, a similar collagenized and detached layer is best seen with a Masson trichrome stain; there is no biliary epithelium remnant (x10). **c** and **d**. Again, in Case 3, a hyaline membrane appears semi-detached in the cyst lumen, and overlying a cyst wall with vascular congestion and hemorrhagic suffusion, but no epithelium (C, H&E, x100). On higher magnification, the membrane is dense and paucicellular (D, H&E, x200). **e** and **f.** Case 4 showed no obvious cystic dilatation. The common hepatic duct showed a lack of epithelium, the luminal portion of the duct showing fibro-edematous changes (E, Masson trichrome, x100). The inner portion of the ductus choledochus showed heterogeneous, focally increased collagen deposition (*arrows*), but no hyaline membrane (F, Masson trichrome, x200)

### Pathology findings

In all 4 cases, liver biopsy findings were consistent with BA, showing mild portal tract edema and fibrosis, ductular reaction, and major cholestasis with giant cell transformation. In cases 2, 3 and 4, atretic biliary remnants at the porta hepatis resected at portoenterostomy were surrounded by a fibro-edematous and inflammatory cuff. Bile duct and cyst characteristics are summarized in Table 2.

Histological evaluation of the cyst wall showed, in Cases 1, 2 and 3, a striking dense hyalinised and paucicellular layer. This hyaline membrane was seen on the luminal, inner aspect of the cyst (Fig. 3a, Case 1, and was partially detached in cases 2 and 3 (Fig. 3b, c and d). No residual epithelial lining was seen. In addition, the cyst wall in Case 3 showed intense vascular congestion with hemorrhagic suffusion.

The supposed cyst of the porta hepatis in Case 4 laid within the ductus choledochus. Histology showed a choledochus with a residual lumen of 0.1 mm, with subtotal epithelial loss. The common hepatic duct and the choledochus showed loose fibro-edematous changes, without well-defined hyalinized layer or membrane. Instead, focally increased collagen deposition was seen (Fig. 3e and f).

**Table 2:** Main histological findings

	Case 1	Case 2	Case 3	Case 4
Size of proximal common hepatic duct (mm)	5	3	Atrophic	1.5
Size of hepatic hilar biliary structures (µm)	n.a.	75	50	15–40
Cyst size (cm)	3.5 × 2.5	4	2.5	n.a.
Hyaline membrane	Yes	Yes Partially detached	Yes Partially detached	No Focal collagen deposition
Epithelial lining	None	None	None	Subtotal loss

### Postoperative follow-up

Case 1and 4 cleared jaundice within the first two postoperative weeks. Because of persistent postoperative cholestasis without bile duct dilatation and pathology suggestive of BA, Case 2 underwent a Kasai HPE six weeks after the initial surgery without further supplementary investigations such as hepatobiliary iminodiacetic acid (HIDA) scan and MRCP given the high suspicion of BA based on the pathological findings. We thus decided against additional imaging to avoid delays in the necessary surgical intervention. It is a matter of debate if Case 1 is not a choledochal cyst but a cystic BA.

Cases 1and 2 cleared jaundice and are well and thriving at the current age of 32 and 22 months.

While Case 4 initially followed a similar course, she then developed bile lakes and intermittent cholestatic jaundice. She actually cleared jaundice.

Case 3 presented an early episode of cholangitis at day seven with persistent jaundice and underwent liver transplantation at the age of 8 months (Table 1).

## Discussion

### Pre- and postnatal evaluation

A prenatally discovered hepatic hilar cyst, in the presence of a visible gallbladder, has two distinct differential diagnoses, BA versus CC. There are two widely accepted indicators which may predict diagnosis: (1) the size of the cyst in BA is reported to be smaller than in CC, probably due to the smaller volume of excreted bile [7, 16], and (2) in the case of CC the size of the cyst usually increases continuously before birth [17]. Nonetheless, postnatal diagnosis cannot be readily defined prenatally and postnatal imaging can be equivocal when not able to rule out BA. As the two diagnoses have quite different management and, importantly, a distinctly different prognosis, prenatal counselling of parents is challenging. A further indicator could be abnormal levels of amniotic fluid digestive enzymes measured before 22 weeks of gestation, which have been shown to predict BA in up to 90% of patients with prenatal non-visualization of the gallbladder [18]. However, to the best of our knowledge, the profile of amniotic fluid digestive enzymes has not been analyzed in the presence of prenatal hepatic hilar cysts to confirm or exclude BA.

After birth, the hepatic hilar cyst and associated abdominal features should be investigated by US. In the presence of a hepatic hilar cyst, US features highly suggestive of BA include the triangular cord sign, a small and irregular gallbladder, and abdominal heterotaxia with polysplenia [19]. Further and understandably, the presence of dilated intrahepatic bile ducts is associated with CC and thus rules out BA [20]. Of note, multiple cut-off values on the post-natal US have been identified to differentiate between CC and BA, ranging from 1 cm to 2.5 cm [21, 22]. Consequently, in the 90ies Redkar et al. proposed an algorithm for the management of prenatally diagnosed hepatic hilar cysts based on dilated or non-dilated intrahepatic bile ducts [23]. However, this management algorithm proved to be difficult to apply, since the majority of the patients will not have dilated intrahepatic bile ducts. Last but not least, hepatobiliary scintigraphy used in this particular situation has proven to be misleading because of numerous false-positive findings [24].

**Fig. 4:**
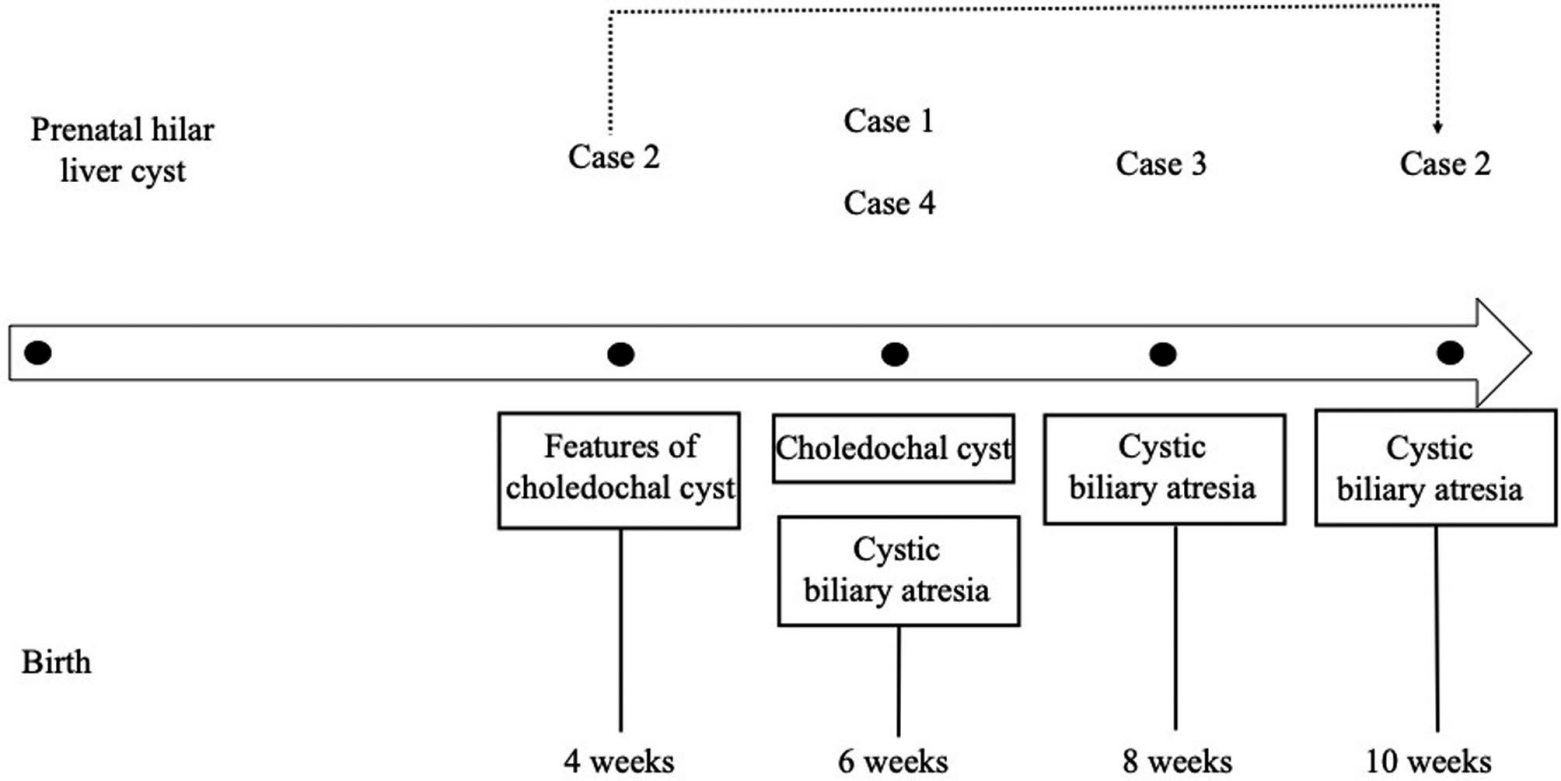
Timeline for final postnatal diagnosis of four patients diagnosed with prenatal hilar liver cysts.

As for biological markers, it appears that newborns with BA present with higher serum aminotransferases, higher total bile acids and increased levels of bilirubin, when compared to babies with CC [16, 20]. Nevertheless, gamma-glutamyl transferase levels were not different in a clinically controlled study comparing CCs with BA [20]. Regular blood work-up might help distinguish between BA and CC patients.

Patients diagnosed with prenatal hepatic hilar cysts with highly suggestive features of BA on US need an early Kasai operation. For a CC not developing jaundice and discolored stool in the postnatal evaluation, the optimal timing for its treatment has not been definitely established yet [7]. That said, it is now accepted that patients have to be monitored closely by US and liver function tests. MRCP is usually only performed around three months to better delineate the anatomy of the cyst and bile ducts before elective surgery, scheduled between 3 and 6 months to avoid complications [25]. A meta-analysis revealed 68% positive predictive value and 94.3% negative predictive value of the MRI in diagnosing BA [26]. The effectiveness of MRCP in reliably excluding BA at the optimal time of surgical intervention is limited by the non-visualisation of the extrahepatic bile ducts in 37.5% of infants less than 3 months of age under normal physiologic conditions and 50% of infants less than one month [27]. A HIDA scan can be proposed for the pre-operative work-up; absent excretion of radiolabeled contrast into the duodenum raises the suspicion of biliary atresia [28, 29]. The same study identified 64.5% positive predictive value and 97.2% negative predictive value for the HIDA scan in diagnosing BA; thus, besides its irradiation, false positive results might occur in patients with medical cholestasis [26]. The “intermediate cases” who develop jaundice should undergo prompt IOC [7] to differentiate an “obstructive” CC from cystic BA [30]. It is of the utmost importance that the patient be transferred to a center specialized in the management of BA.

### Summary and recommendation for pre- and postnatal evaluation (Fig. 5)

Foetuses with a prenatally diagnosed hepatic hilar cyst in the presence of a visible gallbladder need early postnatal evaluation with US. Cases where the diagnosis remains unclear and CC or BA cannot be confirmed by postnatal US, need close observation. Cholestasis must lead to early IOC for a timely Kasai.

### Intraoperative cholangiogram and surgery

IOC is the current gold standard for diagnosing BA. Cholangiography patterns (evaluation of the intrahepatic bile ducts and duodenal communication) can differentiate classical CC and BA patients [31].

During cholangiography, even in patients with a CC, the duodenal communication is not always visible and biliary fluid coming from patent intrahepatic bile ducts can be clear due to longstanding extrahepatic biliary obstruction by a blind-ending CC [32]. Similar cases to our Case 1 were reported with a lack of distal communication with the duodenum, a finding usually associated with BA [9, 32].

A description of the IOC patterns of the proximal bile ducts for cystic BA includes (i) cloudy, (ii) tree-like or (iii) mixed pattern. These three findings have been shown to be associated with outcomes for native liver survival of 50%, 100% and 78%, respectively [3]. It has been proposed to perform a hepatico-jejunostomy for patients with a hepatic duct diameter ≥ 1 mm [4], or whenever the duct seemed large enough to accommodate an anastomosis [33]. Jaundice clearance rates were significantly improved also whenever the hepatic radicles have a diameter of ≥ 1 mm when compared to < 1 mm diameter (93% vs. 77%) [4].

Cyst amylase and lipase concentration is usually analyzed, with high values speaking in favor of a pancreaticobiliary malunion and thus a CC [34]. However, it has been stipulated that pancreaticobiliary malunion explains CC in older patients [35], while neonatal forms of CC are believed to be “congenital”. They rather seem to present a weakness of the common bile duct in its early developmental stages, allowing its dilatation when intraductal pressure increases due to obstruction of the distal common duct [36]. Furthermore, pancreatic enzyme activity seems to increase only after one month of extrauterine life [22]. It is thus not surprising that Case 1 did not show increased values of pancreatic enzymes in the cyst fluid.

While the management of CC is unequivocal with cyst excision and hepatico-jejunostomy, the best surgical strategy for cystic BA is still debated. Historically, a Kasai HPE was proposed for cystic BA [37]. Native liver survival for patients with cystic BA and Kasai has been shown to be 68% with a patient survival of 76% (Table 3). Later, hepatico-jejunostomy has been described for cystic BA [4, 33, 38]. Excellent results have been reported for both open and laparoscopic hepatico-jejunostomy performed for cystic BA [5], [39], with 98% patient survival and 92% native liver survival (Table 4). The reported literature consists mainly of case reports with very different follow-up periods. Our findings suggest a possible benefit in native liver and patient survival from performing a hepatico-jejunostomy for cystic BA, although these outcomes are influenced by multiple factors including the timing of the surgery, bile duct diameter, postoperative care, etc., factors that are not always well documented in the below mentioned studies.

**Table 3:** Outcome of Kasai hepato-porto-enterostomy for cystic biliary atresia

Article	# of patient	Age at Kasai HPE (days)	Outcome	Follow-up (years)
Komuro H [5]	2	54 57	Jaundice free Jaundice free	20 7.7
MacKenzie T [40]	1	11	Jaundice free	0.8
Masumoto K [41]	1	30	Jaundice free	n.a.
Sinha S [42]	1	45	Dead (cholangitis)	0.25
Suzuki T [12]	1	203	Jaundice free	17
Fatahi N [28]	1	n.a.	Jaundice free	0.8
Tanaka H [7]	3	14 50 48	Jaundice free	2.1
Nio M [4]	26	n.a.	8 Dead 3 LT	n.a.
Koshinaga T [43]	1	120	Jaundice free	3
Rahamtalla D [44]	1	56	Jaundice free	n.a.

Kasai HPE, Kasai hepato-porto-enterostomy; LT, liver transplantation

**Table 4:** Outcome of hepatico-jejunostomy for cystic biliary atresia

Article	# of patient	1st surgery	1st surgery age (days)	Nr. (%) Kasai HPE	Outcome	Follow-up (years)
Tsuchida Y [45]	1	HJ	23	n.a.	Jaundice free	1.5
Matsubara H [46]	1	HJ	22	n.a.	Jaundice free	0.5
Komuro H [5]	1	HJ	30	n.a.	Jaundice free	17
	1	HJ	25	n.a.	Jaundice free	2.5
	1	HJ	152	n.a.	LT	0.8
	1	HJ	84	n.a.	Jaundice free	0.3
Nio M [3]	22	HJ	n.a.	3/22 (13.6%)	n.a.	n.a.
Caponcelli E [2]	1	HJ	n.a.	1	Jaundice free	n.a.
Takahashi Y [33]	12	HJ	71.6 (24–136)	1/12 (8.3%)	9/12 (75%) Jaundice free 1 Recurrent jaundice 2 LT	1.1–22
Faure A [47]	3	Laparoscopic HJ	n.a.	n.a.	2 Jaundice free 1 Recurrent jaundice	5.1
Nio M [4]	14	HJ	n.a.	3/14 (21.4%)	10/14 (71.4%) Jaundice free 2 LT 2 Dead	n.a.
Lal R [38]	2	HJ	32 45	n.a.	Jaundice free Jaundice free	n.a.
Ji Y [39]	27	Laparoscopic HJ	67.9+/- 35.6	n.a.	25/27 (92.6%) Jaundice free	6+/-2.9
Calinescu AM, 2024 (actual series)	1	HJ	28	1	Jaundice free	1.8

HJ, hepatico-jejunostomy; Kasai HPE, Kasai hepato-porto-enterostomy; LT, liver transplantation

### Summary and recommendation for cholangiography and surgery (Fig. 5)

**Fig. 5:**
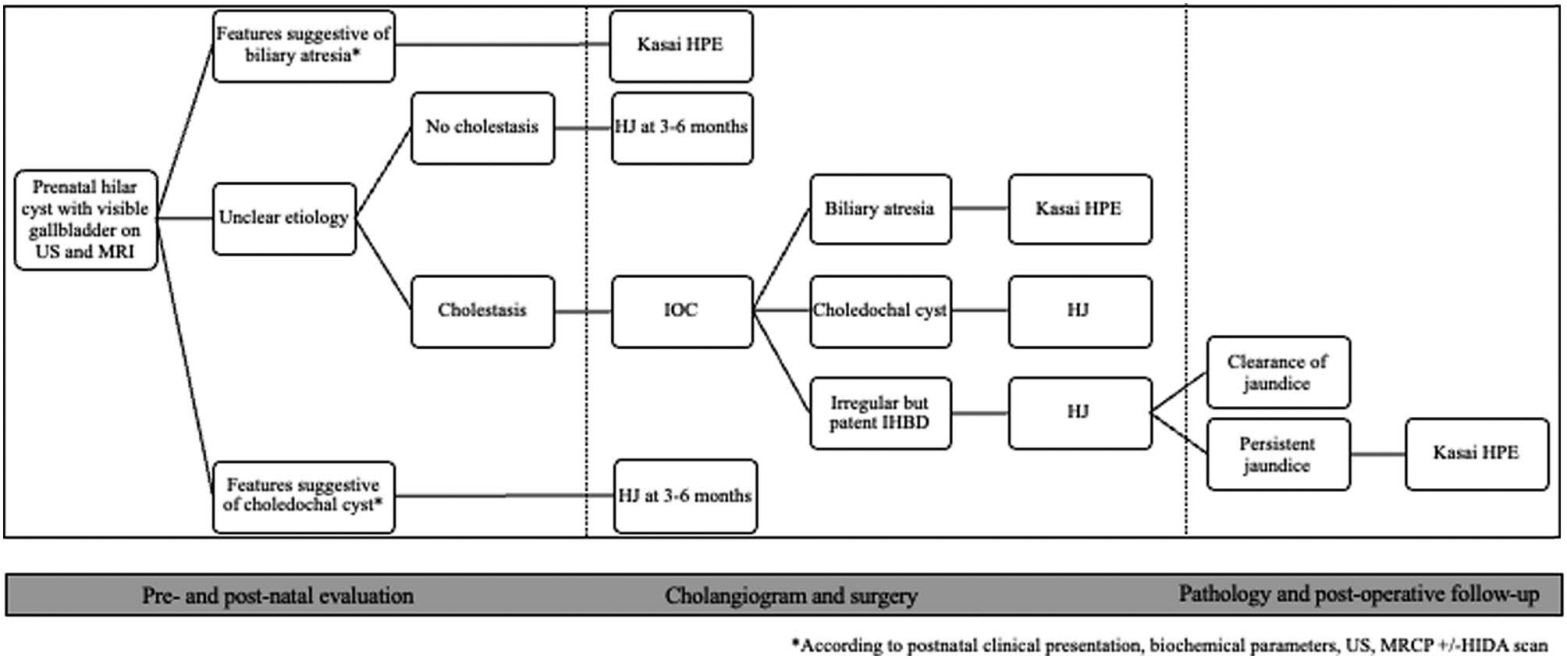
Algorithm for the management of prenatal hepatic hilar cysts in the presence of a visible gallbladder. IOC, intraoperative cholangiogram; HPE, hepato-porto-enterostomy; IHBD, intrahepatic bile ducts; HJ, hepaticojejunostomy; MRI, magnetic resonance imaging; US, ultrasound

IOC is the key step in determining surgical treatment. In the case of an obvious CC an hepatico-jejunostomy is performed. If no intrahepatic bile ducts are seen, a standard Kasai must be performed. In the case of irregular intrahepatic bile ducts and/or lack of communication with the duodenum, if macroscopic intrahepatic bile ducts are seen, it is reasonable to perform a hepatico-jejunostomy for jaundice clearance.

### Pathology findings

A common physiopathology of infantile obstructive cholangiopathy in BA and CC was suggested for the first time by Landing in 1974 [48]. The recent demonstration of the loss of primary cilia from cholangiocytes in both BA and CC, together with the presence in a subset of cases of a ductal plate malformation or of laterality defects, have led to the proposal to include these conditions in the spectrum of ciliopathies [49].

A complete or near complete lack of a biliary type epithelial lining has been reported in the cysts of BA as opposed to CC [2, 50, 51]. Accordingly, all four cases reported here showed total or subtotal absence of an epithelial lining.

The more recent descriptions highlight the presence of a striking grossly visible whitish membrane corresponding upon histological evaluation to an inner densely collagenized layer that tended to separate from the outer portion of the cyst wall [50, 51]. It is speculated that this membrane may be due to subepithelial degenerative changes, perhaps related to the accumulation of fluid [51]. In the series by Lobeck et al. [50]. no subluminal hyaline layer was seen in 18 of the 21 CC reported cases. In the remaining patients, a layer of sclerotic collagen was observed. These three infants had liver biopsy features of obstructive cholangiopathy; however a diagnosis of BA was not retained at surgery and follow-up [50]. This highlights the potential morphological overlap between BA and CC. A similar paucicellular hyalinized membrane was observed in Cases 1 to 3. Both the hepatic duct and the choledochus in case 4 showed only focal areas of increased collagen deposition. The resection specimen showed no obvious cystic dilatation, and this case may represent a more frustre form of cystic BA.

Liver histology may also reveal differences between the two entities, with portal tract fibrosis and features of chronic cholangiopathy tending to be more severe in BA than in CC [51], although significant overlap can be seen [31, 49]. It can be argued if a needle liver biopsy should be obtained prior to IOC for these patients. Nevertheless, if patients present with jaundice and discolored stool a prompt intervention is warranted. If the pathology results can influence the further course of action, the first intervention performed for these children is not influenced by the pathology results.

### *Summary and recommendation for pathology findings*

Pathology is essential to guide follow-up in patients with hepatic hilar cysts and irregular intrahepatic bile ducts who develop obstructive jaundice. In case of histology compatible with BA, patients previously identified as presenting CC, should be monitored closely.

### Postoperative follow-up

The chronology of neonates with prenatal hepatic hilar cyst has shown that a subset of patients will evolve from a cystic BA with patent but abnormal intrahepatic bile ducts, to BA with atretic bile ducts proximal to the porta hepatis [41]. Further, another physiopathological correlation was found between an extrahepatic biliary cyst perforation and a cystic BA with atretic bile ducts proximal to the porta hepatis [43]. Two explanations were proposed: first, overlapping features of CCs and BA might be due to the young age of the patients, more than to the BA per se; second, the early presentation of some cystic BA patients might be the same as CC [50]. It was also hypothesized that patients with CCs or cystic BA might progress to type III BA [2, 6, 12, 41, 43]. The course of Case 2 supports the hypothesis of an evolutive process between the CC and cystic BA. One might postulate that a continuum exists in the development of CCs, cystic BA with patent but abnormal intrahepatic bile ducts, to BA with atretic bile ducts proximal to the porta hepatis (Fig. 4). Therefore, it is not surprising that the Kasai procedure has shown excellent results in patients after an unsuccessful hepatico-jejunostomy performed for cystic BA (Table 2).

### Summary and recommendation for postoperative follow-up (Fig. 5)

In patients with an antenatal history of a hepatic hilar cyst who develop obstructive jaundice and presented with irregular bile ducts on IOC at the time of hepatico-jejunostomy, careful follow-up is essential to convert to Kasai HPE in a timely fashion.

### Strengths and limitations

While this case series provides valuable insights into the challenging scenario of prenatally diagnosed hepatic hilar cysts, its findings should be interpreted with consideration of the small sample size, retrospective nature, and the need for caution in managing transitional types. Further research is warranted to validate the proposed management algorithm and enhance the understanding of this complex clinical scenario.

## Conclusions

The management of prenatally diagnosed hepatic hilar cysts in the presence of a visible gallbladder is challenging. A management strategy was developed to help clinicians to navigate this situation (Fig. 5). Even if IOC differentiates most patients with BA from those with a CC, for hybrid indeterminate forms with irregular intrahepatic bile ducts, caution is advised. A hepatico-jejunostomy can be performed for these forms, but the patient needs a close, specialized follow-up, as a secondary Kasai HPE might be required.

## Data Availability

No datasets were generated or analysed during the current study.

## Abbreviations

BA: Biliary atresia
CC: Choledochal cyst
US: Ultrasound
IOC: Intraoperative cholangiogram
HPE: Hepato-porto-enterostomy
HIDA: Hepatobiliary iminodiacetic acid scan
MRCP: Magnetic resonance cholangiopancreatography
